# Dissection of blood–brain barrier dysfunction through CSF PDGFRβ and amyloid, tau, neuroinflammation, and synaptic CSF biomarkers in neurodegenerative disorders

**DOI:** 10.1016/j.ebiom.2025.105694

**Published:** 2025-04-15

**Authors:** Agathe Vrillon, Nicholas J. Ashton, Elodie Bouaziz-Amar, François Mouton-Liger, Emmanuel Cognat, Julien Dumurgier, Matthieu Lilamand, Thomas K. Karikari, Vincent Prevot, Henrik Zetterberg, Kaj Blennow, Claire Paquet

**Affiliations:** a Cognitive Neurology Centre, Lariboisière Fernand Widal Hospital, Assistance Publique Hôpitaux de Paris, Université Paris Cité, Paris, France; b INSERM U1144, Therapeutic Optimization in Neuropsychopharmacology, Paris, France; c University of California San Francisco, San Francisco, CA, USA; d Department of Psychiatry and Neurochemistry, Institute of Neuroscience and Physiology, The Sahlgrenska Academy at the University of Gothenburg, Mölndal, Sweden; e Banner Alzheimer's Institute and University of Arizona, Phoenix, AZ, USA; f Banner Sun Health Research Institute, Sun City, AZ 85351, USA; g Centre for Age-Related Medicine, Stavanger University Hospital, Stavanger, Norway; h Biochemistry Department, AP-HP. Nord, Site Lariboisière Fernand-Widal, Paris, France; i Department of Psychiatry, University of Pittsburgh, Pittsburgh, PA, USA; j Univ. Lille, Inserm, CHU Lille, Laboratory of Development and Plasticity of the Neuroendocrine Brain, Lille Neuroscience & Cognition, UMR_S1172, DISTALZ, Lille, France; k Clinical Neurochemistry Laboratory, Sahlgrenska University Hospital, Mölndal, Sweden; l UK Dementia Research Institute at UCL, London, UK; m Hong Kong Centre for Neurodegenerative Diseases, Clear Water Bay, Hong Kong Special Administrative Region of China; n Wisconsin Alzheimer's Disease Research Centre, University of Wisconsin School of Medicine and Public Health, University of Wisconsin–Madison, Madison, WI, USA; o Paris Brain Institute, ICM, Pitié-Salpêtrière Hospital, Sorbonne University, Paris, France; p Neurodegenerative Disorder Research Centre, Division of Life Sciences and Medicine, and Department of Neurology, Institute on Aging and Brain Disorders, University of Science and Technology of China and First Affiliated Hospital of USTC, Hefei, PR China

**Keywords:** Brain blood barrier, Neurodegenerative disorders, Neuroinflammation, Alzheimer's disease, CSF biomarkers, PDGFRβ

## Abstract

**Background:**

Blood–brain barrier (BBB) dysfunction is an early event in neurodegenerative disorders. Pericytes are key cells for BBB maintenance. Upon pericyte injury, the platelet-derived growth factor receptor-β (PDGFRβ) is released in the cerebrospinal fluid (CSF). The relation of CSF PDGFRβ with markers of amyloid pathology, neuroinflammation, and axonal and synaptic damage across dementia remains unclear.

**Methods:**

Retrospectively, we quantified CSF PDGFRβ and CSF core Alzheimer's disease (AD), astrocytic (GFAP), microglial (sTREM 2, YKL-40), axonal (NfL), and synaptic (GAP-43, neurogranin) biomarkers in 210 patients from the Cognitive Neurology Centre, Paris, France, including n = 23 neurological controls (NC), n = 84 patients with mild cognitive impairment (MCI) [AD, n = 41; non-AD, n = 43], and n = 103 patients with dementia (AD, n = 73; non-AD, n = 30).

**Findings:**

Comparing clinical stages, CSF PDGFRβ levels were increased at the MCI stage (Cohen's *d* = 0.55 [CI_95%_ 0.066, 1.0], P = 0.025) compared with NC. Non-AD MCI displayed higher levels than controls (Cohen's *d* = 0.74 [CI_95%_ 0.22, 1.3], P = 0.042). No association was observed with CSF Aβ42/Aβ40 ratio but with p-tau 181 (β = 0.102 [CI_95%_ 0.027, 0.176], P = 0.0080) and t-tau levels (β = 0.133 [0.054, 0.213], P = 0.0010). CSF PDGFRβ levels were positively associated with CSF neuroinflammation and synaptic markers levels. Higher CSF PDGFRβ levels were associated with lower MMSE scores at MCI (β = −1.23 [CI_95%_ −2.33, −0.260], P = 0.015) and dementia stages (β = −2.24 [CI_95%_ −3.62, −0.85], P = 0.0020). CSF neuroinflammation biomarkers mediated the association of CSF PDGFRβ with neurodegeneration and synaptic integrity markers.

**Interpretation:**

CSF PDGFRβ, a candidate biomarker of BBB dysfunction, is increased in the early stages of neurodegenerative disorders, in association with neuroinflammation and axonal and synaptic damage.

**Funding:**

Association des Anciens Internes des Hôpitaux de Paris, Edmond de Rothschild Program, 10.13039/100014808Fondation Vaincre Alzheimer, 10.13039/100010773Demensförbundet, Gamla Tjänarinnor, 10.13039/100010107Anna-Lisa och Bror Björnssons Stiftelse.

**Research in context**

**Evidence before this study**

Blood–brain barrier (BBB) dysfunction has been identified as a key mechanism in various neurodegenerative disorders. However, the exact characteristics of the BBB impairment, including its primary drivers and its temporality along neurodegeneration, remain unclear. Pericytes, a key cellular component of BBB, have been demonstrated to be altered in dementia, including AD. This alteration is considered to be reflected by the release of platelet-derived growth factor receptor-β (PDGFRβ) in the CSF.

**Added value of this study**

We measured CSF PDGFRβ levels, with CSF AD core biomarkers and neuroinflammation and synaptic markers, in a cohort including patients with AD and non-AD dementia. We found that CSF PDGFRβ levels were increased at the MCI stage but were not associated with CSF markers of amyloid pathology. CSF PDGFRβ displayed a significant positive association with markers of neuroinflammation and synaptic markers. Higher levels of CSF PDGFRβ were correlated with poorer cognitive status. Microglial markers mediated CSF PDGFRβ effects on markers of neurodegeneration and synaptic integrity, suggesting a bidirectional relationship between BBB dysfunction and neuroinflammation.

**Implications of all the available evidence**

Our findings bring additional evidence on pericyte dysfunction and BBB impairment as shared mechanisms of disease progression in neurodegenerative disorders. These processes can be monitored by CSF analysis. The inclusion of biomarkers of BBB impairment could contribute to the understanding of cognitive decline and, potentially, in a long-term perspective, in tailored therapy choices.

## Introduction

The blood–brain barrier (BBB) is a specialised brain endothelial structure that functions as a critical diffusion barrier within the central nervous system (CNS).^1^ This barrier is formed by endothelial cells and is supported by adjacent cell types, including pericytes, astrocytes, and microglia, collectively referred to as the neurovascular unit.^2^ The integrity of the BBB is essential for maintaining CNS homoeostasis, including the proper functioning of neuronal circuits, synaptic transmission and remodelling, angiogenesis, and waste clearance. By restricting neuronal contact with circulating toxins, pathogens, and inflammatory molecules, the BBB plays a pivotal role in preserving neuronal function and mitigating neuroinflammation.

BBB dysfunction has been associated with neurodegeneration and ageing.^3^^,^^4^ In Alzheimer's disease (AD), there is significant evidence of a disruption of the BBB homoeostasis through a bidirectional association.^5^ Neurotoxic accumulation of amyloid beta (Aβ) is hypothesised to contribute to the dysfunction of the BBB, which will, in turn, aggravate Aβ deposition. There is also evidence that BBB impairment could contribute to disease in the spectrum of synucleinopathies.^6^ BBB alterations have been documented through the measure of albumin quotient (Q-Alb), S100 calcium-binding protein B (S100B), and neuron-specific enolase (NSE).^7^^,^^8^ Imaging studies using high spatial and temporal resolution techniques such as dynamic enhanced MRI have also demonstrated loss of BBB integrity.^9^

Platelet-derived growth factor (PDGF) signalling regulates various functions in the CNS, including neurogenesis, cell survival, and synapse plasticity, across multiple brain cell types.^10^ Both vascular and astroglial cells at the BBB express PDGF ligands as well as receptors.^11^^,^^12^ Among those receptors, platelet-derived growth factor receptor beta (PDGFRβ) is a tyrosine kinase receptor predominantly expressed in pericytes, with some expression also present in vascular smooth muscle cells, and vascular-associated and meningeal fibroblasts.^13^ Pericytes are perivascular cells contributing to vessel stability through mechanical stabilisation and signalling toward endothelial cells and astrocytes.^14^^,^^15^ PDGFRβ promotes pericyte recruitment during angiogenesis and is involved in the maintenance of cerebral microcirculation at the BBB.^16^ PDGFRβ signalling has been described as altered in some neurodegenerative disorders, including AD and synucleinopathies.^17^^,^^18^ In AD, modifications of the abundance of PDGFRβ and of its ligand PDGF-BB have been reported, albeit inconsistently, across brain regions.^17^^,^^19^ PDGFRβ is released upon pericyte injury and can be measured in CSF. Increased levels have been reported in AD and some non-AD dementias.^19^, ^20^, ^21^, ^22^, ^23^ However, the association with markers of amyloid and tau pathology and the link to clinical syndromes remain unclear. Additionally, evidence is still lacking in non-AD dementia.

In this study, we aimed to: (i) measure CSF levels of PDGFRβ in a cohort comprising subjects with AD and non-AD dementia; (ii) explore the association between PDGFRβ and established biomarkers of amyloid, tau, neuroinflammation, and synaptic impairment to investigate the interplay between neurodegeneration, neuroinflammation, and BBB impairment; and (iii) investigate the relationship between these markers and cognitive status.

## Methods

### Cohort

We performed a retrospective observational biomarker study. We systematically included patients seen for a cognitive complaint or decline who had undergone CSF analysis at the Centre of Cognitive Neurology at Lariboisière University Hospital, Paris, France, between March 2014 and December 2019, with available CSF and plasma samples through the BioCogBank protocol (NCT06244875, Biological Collection of Neurocognitive Disorders). The cohort (total n = 210) encompassed neurological controls (NC, n = 23), and patients with non-AD mild cognitive impairment (non-AD MCI, n = 43), non-AD dementia (n = 30), AD-MCI (n = 41), and AD dementia (n = 73). Patients with non-AD dementia included patients with dementia with Lewy bodies (DLB, n = 12), frontotemporal dementia (FTD, n = 13), vascular cognitive impairment, and dementia (VCID, n = 4) and Creutzfeldt Jakob disease (CJD, n = 1). Patients underwent neurological clinical examination, neuropsychological assessment, *APOE* genotyping, brain magnetic resonance imaging (MRI), clinical blood and CSF analysis, and fluid sampling for research (blood and CSF). Sex was self-reported by participants. Race and ethnicity were not collected as prohibited by French law. Diagnoses were reviewed during multidisciplinary consensus meetings (including neurologists, neuropsychologists, geriatricians, and biochemists) considering results of validated CSF biomarkers and according to clinical criteria for AD dementia, AD-MCI, DLB, FTD, VCID, and CJD.^24^, ^25^, ^26^, ^27^, ^28^, ^29^ Patients with AD displayed CSF biomarkers on the AD continuum, i.e., a positive CSF amyloid status.^26^ Non-AD MCI presented with normal CSF biomarkers or suspected non-Alzheimer pathophysiology (normal Aβ42/Aβ40, high p-tau and/or high t-tau). NC were individuals referred for a clinical complaint, presenting with normative or subnormative cognitive testing, no abnormalities on imaging and CSF markers, and no cognitive decline over the follow-up period of several years.

### Sample collection

Lumbar puncture was performed after overnight fasting. CSF samples were centrifuged at 1000*g* for 10 min at 4 °C within 2 h of collection and then aliquoted into 0.5 mL polypropylene tubes before being stored at −80 °C for further analysis. Blood was sampled in a 3-h window from the CSF sampling. Blood samples were obtained through venipuncture in fasting condition and collected into ethylenediaminetetraacetic acid (EDTA) tubes. Samples were centrifuged at 2000*g* for 20 min at 4 °C. Plasma supernatant was collected and frozen at − 80 °c. Albumin CSF and plasma levels and *APOE* genotype were assessed in clinical practice at the Biochemistry Department, Lariboisière Hospital, Paris, France. All other biomarkers were analysed at the Department of Psychiatry and Neurochemistry, University of Gothenburg, Sweden.

### Biomarker measurements

CSF and plasma PDGFR levels were measured using a commercial ELISA kit validated for (Kit #EHPDGFRB Invitrogen, Thermo Fisher Scientific, Waltham, MA, USA) according to the manufacturer's recommendations. CSF samples (100 μL undiluted) and plasma (diluted 1 in 40) were used for analysis. CSF and plasma from each subject were run on the same plate. Standards, samples, and blanks were added in duplicate. Absorbance was read at 450 nM in a FLUOstar OPTIMA plate reader (BMG labtech, Aylesbury, UK). The inter-assay CV was <12% and the intra-assay was <10%. PDGFRβ concentration in samples was calculated by interpolation against the standard curve for each case, derived from serial dilutions of recombinant PDGFRβ (18,000–24 pg/mL). CSF YKL-40 levels were measured using a commercial ELISA kit from R&D System (Kit #DY2599, Minneapolis, MN, USA). CSF soluble triggering receptor expressed on myeloid cells 2 (sTREM 2) was measured in-house using an electrochemiluminescence immunoassay from Gothenburg University, Sweden with a Meso Scale Discovery (MSD) SECTOR imager 6000 (MSD, Rockville, MD, USA), as described by Alosco et al.^30^ CSF glial acidic fibrillary protein (GFAP) was quantified using the HD-X SIMOA platform using a commercial kit by Quanterix (Kit #Simoa™ GFAP Discovery Kit, Quanterix®, Billerica, MA, USA).^31^ CSF neurofilament light chain (NfL) was measured using an in-house sandwich ELISA from Gothenburg University, Sweden, as described previously.^32^ CSF albumin levels were measured using a turbidimetric assay from Binding Site® (Kit #Optilite assay menu MKG763, Binding Site®, Birmingham, England). Plasma albumin levels were measured using an immunoassay kit from Diagam® (Kit #ALTUR Universal IFU FR v09, Diagam®, Ghislenghien, Belgique) on an Alinity analyser from Abbott® (Chicago, IL, USA). Core AD biomarkers (Aβ40, Aβ42, phospho-tau 181 [p-tau181], and total-tau [t-tau]) were measured on the Lumipulse G1200 platform (Kits #G β-Amyloid 1–42 CSF, G β-Amyloid 1–40 CSF, G pTau 181 CSF, G Total Tau, Fujirebio®, Malvern, PA, USA). The following cut-offs were applied for positivity: Aβ42/Aβ40 < 0.61 pg/mL; p-Tau 181 > 61 pg/mL; t-tau >450 pg/mL.^33^ CSF profile was classified according to the ATN classification: A+ indicating positive Aβ42/Aβ40 ratio, T ± indicating positive p-tau181, and N+ indicating positive t-tau levels.^26^

*APOE* genotype was available for all patients, established using denaturing high-performance liquid chromatography (WAVE® DNA fragment analysis system, Transgenomic, Omaha, NE, USA) after amplification of exon 4 of the *APOE* gene.

### Statistics

The cohort was analysed in clinical syndromes (NC, MCI all causes, dementia all causes) and in diagnosis groups (NC, AD-MCI, AD dementia, non-AD MCI, and non-AD dementia). Demographic data were presented as median [IQR] for continuous variables and % (number of subjects) for categorical variables. Continuous variables were compared across groups using the Kruskal–Wallis test, and categorical variables using Fisher's exact test. All biomarker values were log-transformed before analysis. We excluded outliers defined by a value >mean ± 3SD, including 4 values for plasma PDGFRβ (n = 1 non-AD MCI, n = 2 AD dementia, n = 1 non-AD dementia) and 4 values for the plasma/CSF PDGFRβ ratio (n = 3 AD dementia, n = 1 non-AD dementia). No outlier was observed for CSF PDGFRβ.

We used a direct acyclic graph (DAG) to identify potential confounders using the modified disjunctive cause criterion.^34^ While temporality cannot be definitively established in a cross-sectional design, this approach helps visualise the assumed structure of associations. The DAG was constructed to focus on the primary relationship of interest, the effect of blood barrier impairment reflected by CSF PDGFRβ on the cognitive status (NC, MCI, or dementia). The minimal sufficient adjustment set included age, sex, and education levels. APOE4 status was not included as a confounder as we considered from previous literature that we could not exclude that it could be causal to the exposure. Biomarker levels were compared across groups using one-way ANCOVA with adjustment for our set of covariates, followed by post hoc Tukey's test with adjustment for multiple comparisons. Effect sizes were estimated with Cohen's *d*. Assumptions of normality, homogeneity of variances, linearity, and homogeneity of regression slopes have been checked prior to analysis. We calculated that our sample would allow the detection of a small effect size difference of f = 0.2 with a power of 80% and a two-sided alpha of 5% between 3 groups, adjusting on 2 covariates.^35^

The association of CSF PDGFRβ with CSF biomarkers was studied unadjusted using Spearman's rank correlation and adjusted using linear regression adjusting for age, sex, and CSF Aβ42/Aβ40 ratio. The association of CSF PDGFRβ with MMSE was examined with linear regression models adjusted for age, sex, and levels of education. Model-specific assumptions were checked for all regressions. The normality of residuals was examined by visual inspection of the histograms and using normal probability plots (p–p plots). The homogeneity of variance of residuals was examined by plotting the residuals against the fitted values. The linearity of quantitative predictors with the dependent variable was visually confirmed. Collinearity was excluded with the Variance Inflation Factor (VIF inferior to 4).

Principal component analysis (PCA) was performed on the whole cohort and in the clinical syndrome groups (MCI, dementia) to explore the association between the different biomarkers. Biomarker values were standardised as z-scores before analysis. The suitability of the dataset was evaluated by the Kaiser–Meyer–Olkin Measure of Sampling Adequacy test and Bartlett's Test of Sphericity. The analysis was not performed in the NC as the group did not meet sample adequacy criteria. The number of components was determined by the number of eigenvalues greater than one. A Varimax rotation was performed. Variables with a loading factor >0.4 or < −0.4 were regarded as representative of the component.^36^

We employed simple mediation models to examine the mediation effect of neuroinflammation markers in the association of CSF PDGFRβ with markers of neurodegeneration and synaptic integrity. Analysis was performed using SPSS PROCESS macro version 4.2. The mediation analysis included the model predicting the mediator (CSF YKL-40 or CSF sTREM 2) from the independent variable (CSF PDGFRβ) and the model predicting the dependent variable (CSF t-tau, CSF GAP-43, or CSF neurogranin) from the independent variable (CSF PDGFRβ) and a mediator (CSF YKL-40 or CSF sTREM2). Age, sex, and APOE4 status were added as covariates. The natural indirect, direct, and total effects were estimated by computing unstandardised estimates and 95% confidence intervals (CI_95%_) using non-parametric bootstrapping with 10,000 replications. The CI_95%_ were derived from the 2.5th and the 97.5th percentiles of the bootstrap estimates. The different model assumptions, including normality of the residuals, homogeneity of variance, and exclusion of collinearity, have been verified and met.

A two-sided P-value <0.05 was considered statistically significant across the analysis. Statistical analysis was performed using SPSS 29.0 IBM (Armonk, NY, USA) and R version 4.1 (https://www.r-project.org/). Graphs were generated using GraphPad PRISM 9.0 (San Diego, CA, USA).

### Ethics

All patients have given written informed consent for their participation in the study. The BioCogBank study, including the collection and analysis of samples, was approved by the ethics committee Comité de Protection des Personnes Est III (2023-A01413-42, 23-10-02) and followed the principles of the Declaration of Helsinki.

### Role of funders

The funders had no role in the study design, data collection, data analysis, interpretation, writing of report, or decision to publish.

## Results

Our study included n = 210 participants (age, median [IQR], 70 [63–77]; male: 39%). Out of the 210 participants, n = 23 were NC, n = 84 presented with MCI, and n = 103 with dementia (Table 1). Patients with MCI and dementia were significantly older than NC (age, median [IQR], respectively 72 [63–77] and 71 [65–77] versus 61 [57–69] years, P = 0.010). Characteristics by diagnosis are presented in Supp. Table S1 and by sex in Supp. Table S2.

**Table 1:** Cohort characteristics and biomarker levels.

	Whole cohort	NC	MCI	Dementia	P-value^a^
**n**	210	23	84	103	
Age, years	70 [63–77]	61 [57–69]	72 [63–77]	71 [65–77]	0.010
Sex, male	39% (82)	39% (9)	37% (31)	41% (42)	0.86
*APOE* ε4 carriership	46% (96)	39% (9)	35% (29)	56% (58)	0.0090
MMSE	24 [19–27]	28 [26–30]	25 [22–27]	20 [17–25]	<0.0001
Education levels, below high school level/above high school level/NA	18% (37)/73% (153)/9% (20)	9% (2)/78% (18)/13% (3)	11% (9)/82% (69)/7% (6)	25% (26)/64% (66)/11% (11)	0.040
**AD CSF biomarkers**					
CSF Aβ40, pg/mL	11,715 [9145–14,414]	11,706 [10,514–13,548]	12,131 [8859–14,771]	11,334 [9007–14,177]	0.55
CSF Aβ42, pg/mL	637 [457–986]	1036 [925–1253]	653 [500–1103]	547 [417–771]	<0.0001
CSFAβ42/40 ratio	0.055 [0.04–0.091]	0.094 [0.087–0.099]	0.0625 [0.042–0.0919]	0.046 [0.038–0.063]	<0.0001
CSF p-tau 181, pg/mL	60.1 [34.5–99.4]	33.4 [26.5–39.1]	54.4 [35.0–81.5]	75.4 [49.8–122.4]	<0.0001
CSF t-tau, pg/mL	411 [282–624]	226 [193–291]	400 [276–565]	524 [329–830]	<0.0001
**ATN status**					<0.0001
A-T-	41% (86)	100% (23)	47% (40)	22% (23)	
A + T-	10% (20)	–	11% (9)	11% (11)	
A + T+	46% (97)	–	38% (32)	63% (65)	
A-T+	3% (7)	–	4% (3)	4% (4)	
**Blood brain barrier biomarkers**					
CSF/plasma albumin quot. (Q-Alb)	6.12 [4.62–7.50]	5.83 [4.22–7.84]	6.46 [5.22–8.46]	4.49 [4.50–7.62]	0.035
CSF PDGFRβ, pg/mL	549 [431–700]	474 [340–596]	588 [444–781]	539 [440–691]	0.027
Plasma PDGFRβ, ng/mL	8.79 [7.03–1.11]	7.50 [5.64–9.42]	9.21 [7.72–10.8]	8.01 [6.45–12.3]	0.69
CSF/plasma PDGFRβ ratio	0.064 [0.046–0.089]	0.061 [0.039–0.082]	0.066 [0.048–0.086]	0.063 [0.047–0.093]	0.80

Abbreviations: Aβ, amyloid beta; *APOE*, apolipoprotein E; CSF, cerebrospinal fluid; LoE, levels of education; MCI, mild cognitive impairment; MMSE, mini-mental state examination; NA, non-available; NC, neurological controls; PDGFRβ, Platelet-derived growth factor receptor beta; p-tau 181, tau phosphorylated at serine 181.

Continuous variables are presented as median [IQR] and categorical data as number (%).

a Age and MMSE scores were compared between NC, MCI, and dementia groups using the Kruskal–Wallis test. *APOE* ϵ4 carriership, sex, levels of education, and AT(N) group frequencies were compared between groups using the Fisher's Exact test. In between groups comparison of biomarker levels was performed using one-way ANCOVA adjusted on age and sex.

CSF PDGFRβ levels showed a positive significant association with age in the whole cohort and in the dementia group after adjustment on sex and *APOE* ε4 status (Supp. Table S3, respectively, β = 0.436 [CI_95%_ 0.087 to 0.784], P = 0.015 and β = 0.632 [CI_95%_ 0.158 to 1.105], P = 0.0090). We found no evidence of association with sex in adjusted or unadjusted analysis (Supp. Table S3). *APOE* ε4 carriership was significantly associated with lower CSF PDGFRβ levels only in the NC group (β = −0.164 [CI_95%_ −0.295 to −0.034], P = 0.016, Supp. Table S3), but there was no evidence of association with *APOE* ε4 carriership in the MCI or the dementia groups.

### PDGFRβ and Q-Alb levels in the study population

**Fig. 1:**
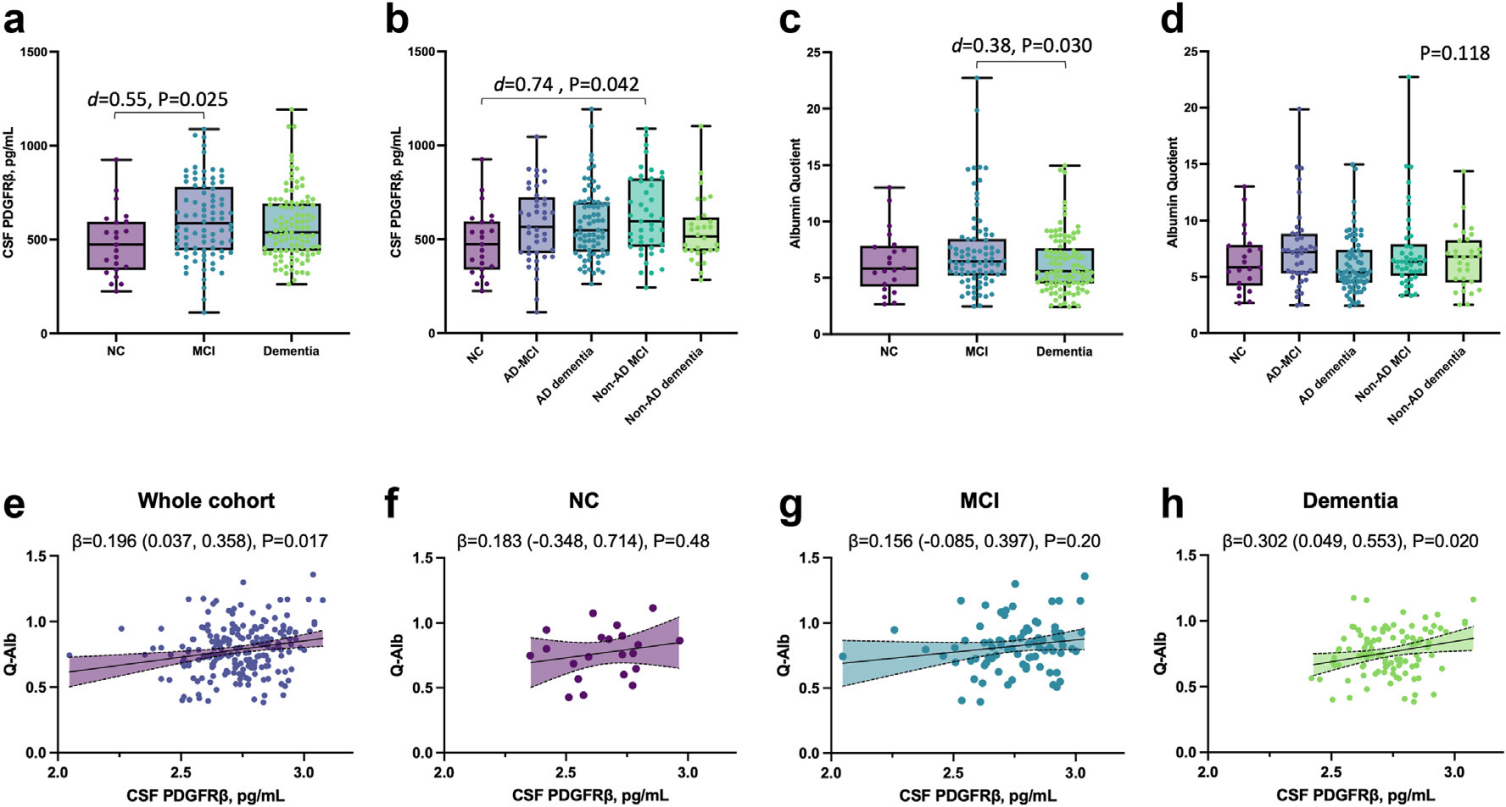
**Association of CSF PDGFRβ and Q-Alb with clinical syndromes and diagnosis**. **a**, CSF PDGFRβ levels across syndrome groups; **b**, CSF PDGFRβ levels across diagnosis groups; **c**, Q-Alb levels across syndrome groups; **d**, Q-Alb levels across diagnosis groups. P-values were obtained through one-way ANCOVA adjusting for age, sex, and levels of education, followed by post hoc Tukey's test, adjusting for multiple comparisons (n = 210). Significant differences (P < 0.05) are reported. Box and whiskers plots with the central line denoting the median value and the box containing the 25th to 75th percentile values. Association of CSF PDGFRβ with Q-Alb, **e**, in the whole sample; **f**, neurological controls; **g**, MCI; and **h**, dementia group. The association between biomarkers was studied using linear regression, adjusting for age and sex (n = 207). Individual points and the regression line are displayed with a shaded area above and below the line, representing the upper and lower bounds of the 95% confidence interval. Results are presented as unstandardised β (95% confidence interval) and P-values.

CSF PDGFRβ levels and Q-Alb values are presented in Fig. 1. Regarding clinical stages, patients at the MCI stage, but not those at the dementia stage, had significantly higher levels of CSF PDGFRβ than NC with a medium effect size (Cohen's d = 0.55 [CI_95%_ 0.066 to 1.0], P = 0.025, Fig. 1a). CSF PDGFRβ did not significantly differ between MCI and dementia groups. Non-AD MCI displayed higher levels than controls with a medium effect size (Cohen's d = 0.74 [CI_95%_ 0.22–1.3], P = 0.042, Fig. 1b). Q-Alb levels were significantly higher at MCI compared with the dementia stage with a small effect size (Cohen's d = 0.38 [CI_95%_ 0.086–0.68], P = 0.026, Fig. 1c). CSF PDGFRβ displayed a weak significant association with Q-Alb in the overall cohort (β = 0.196 [CI_95%_ 0.037–0.358], P = 0.017, Fig. 1e). This significant association was maintained in the dementia group (β = 0.302 [CI_95%_ 0.049–0.553], P = 0.020 Fig. 1h). Association with Q-Alb was not significant in the control and MCI subgroups. There was no evidence of difference found for plasma or CSF/plasma quotient levels for PDGFRβ across groups (Supp. Fig. S2). A weak significant correlation was found between PDGFRβ CSF and plasma levels in the whole cohort (r = 0.154 [CI_95%_ 0.015–0.294], P = 0.027, Spearman's rank test, Supp. Fig. S2), only sustained in the NC group (r = 0.456 [CI_95%_ 0.045–0.722], P = 0.029 Spearman's rank test).

### Association with cognition

CSF PDGFRβ showed a significant weak inverse correlation with MMSE score in the whole cohort (r = −0.164 [CI_95%_ −0.305 to −0.024], P = 0.019, Spearman's rank test, Table 2) as well in the MCI and dementia groups (respectively, r = −0.252 [CI_95%_ −0.468 to −0.018], P = 0.009 and r = −0.211 [CI_95%_ −0.398 to −0.009], P = 0.036, Spearman's rank test). This association remained significant after adjustment for age, sex, and education levels.

**Table 2:** Association of CSF PDGFRβ with cognition.

Whole cohort		NC		MCI		Dementia	
**Unadjusted**							
r (CI_95%_)	P-value	r (CI_95%_)	P-value	r (CI_95%_)	P-value	r (CI_95%_)	P-value
−0.164 (−0.305 to −0.024)	0.019	−0.125 (−0.527 to 0.322)	0.53	−0.252 (−0.468 to −0.018)	0.009	−0.211 (−0.398 to −0.009)	0.036
**Adjusted on age, sex and levels and education**							
β (CI_95%_)	P-value	β (CI_95%_)	P-value	β (CI_95%_)	P-value	β (CI_95%_)	P-value
−1.44 (−2.30 to −0.571)	0.0010	−0.909 (−3.52 to 1.71)	0.47	−1.23 (−2.33 to −0.260)	0.015	−2.24 (−3.62 to −0.85)	0.0020

The association of MMSE scores with CSF PDGFRβ in the whole cohort and the clinical syndromes was studied unadjusted using Spearman's rank test (r) with bootstrapped 95% (CI_95%_) confidence intervals and adjusted using linear regression. For adjusted analysis, data is shown as unstandardised β estimates (CI_95%_) and P-values from linear regression models with CSF PDGFRβ as an independent variable, including age, sex, and LoE (in years) as covariates.

### Association with AD biomarkers

**Fig. 2:**
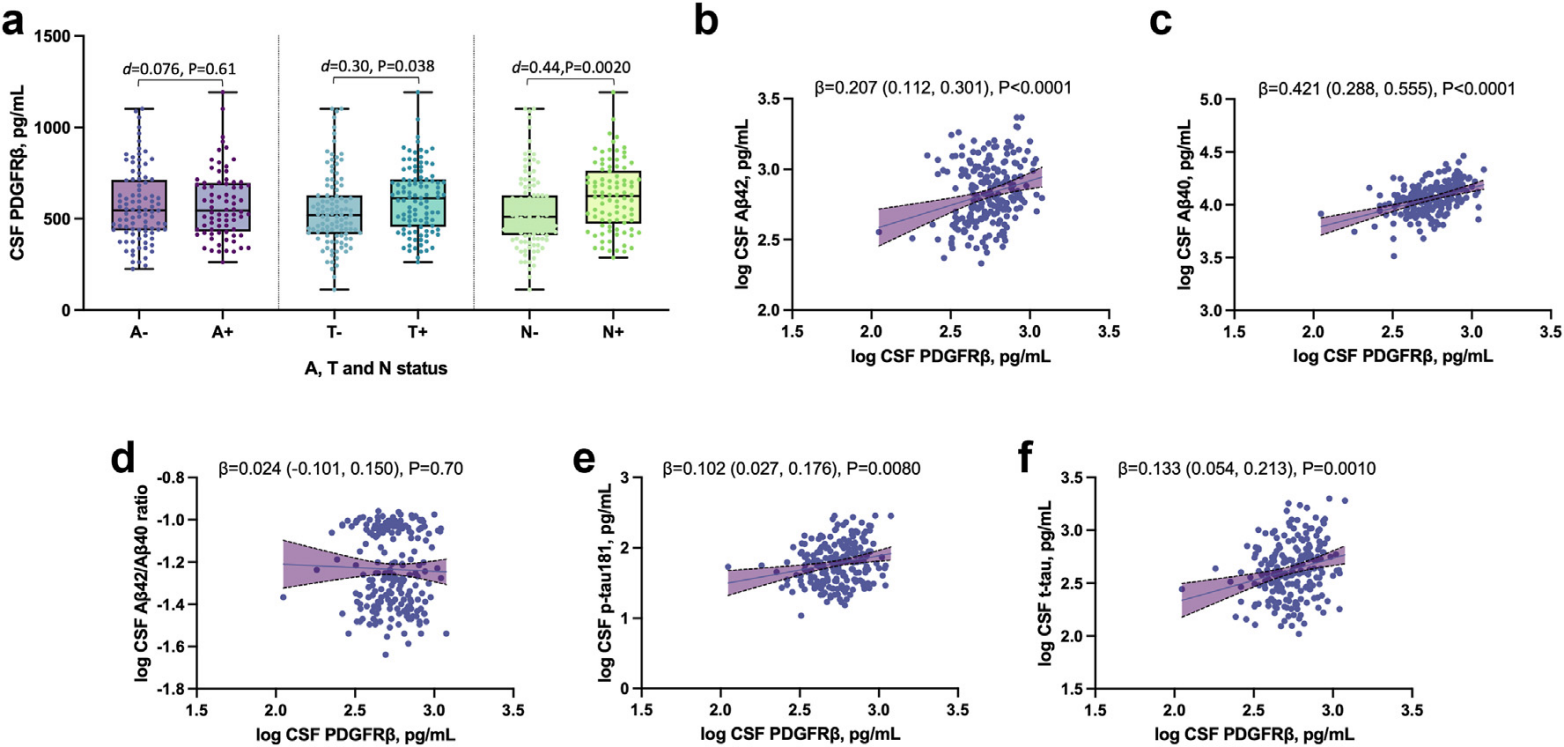
**Association of CSF PDGFRβ with CSF AD biomarkers**. **a**, CSF PDGFRβ levels across AT(N) groups, compared using one-way ANCOVA adjusting for age and sex (n = 210). Box and whiskers plots with the central line denoting the median value and the box containing the 25th to 75th percentile values. **b-e**, Association of CSF PDGFRβ with core AD biomarkers, including **b**, CSF Aβ42; **c**, CSF Aβ40; **d**, CSF Aβ42/Aβ40; **e**, CSF p-tau 181; and **f**, CSF t-tau. The association between biomarkers was studied using linear regression, adjusting for age and sex (n = 210). Individual points and the regression line are displayed with a shaded area above and below the line, representing the upper and lower bounds of the 95% confidence interval. Results are presented as unstandardised β (95% confidence interval) and P-values.

Regarding the association with ATN status across the whole cohort, higher levels of CSF PDGFRβ levels were significantly associated with T positivity (Cohen's *d* = 0.30 [CI_95%_ 0.015–0.59], P = 0.038) and N positivity (Cohen's *d* = 0.44 [CI_95%_ 0.15–0.73], P = 0.0020, Fig. 2a) with small effect sizes for both. Investigating separately MCI and dementia stages, CSF PDGFRβ levels were only significantly higher in the N+ individuals compared to N- at the MCI stage with a medium effect size (Cohen's *d* = 0.50 [CI_95%_ 0.040–0.97], P = 0.031, Supp. Fig. S3a). There was no evidence of difference in CSF PDGFRβ levels between A+ and A-subjects (Cohen's d = 0.076 [CI_95%_ −0.21 to 0.37], P = 0.61, Fig. 2a).

Higher CSF PDGFRβ levels were significantly associated with higher CSF Aβ42 levels (β = 0.207 [CI_95%_ 0.112–0.301], P < 0.0001 Fig. 2b) and Aβ40 levels (β = 0.421 [CI_95%_ 0.288–0.555], P < 0.0001 Fig. 2c). There was no evidence of association with the CSF Aβ42/Aβ40 ratio (β = 0.024 [CI_95%_ −0.101 to 0.150], P = 0.70, Fig. 2d). There was a significant association with CSF p-tau and t-tau levels (respectively, β = 0.102 [CI_95%_ 0.027–0.176], P = 0.0080 and β = 0.133 [CI_95%_ 0.054–0.213], P = 0.0010, Fig. 2e–f).

### Association with CSF neuroinflammation, axonal and synaptic markers

**Fig. 3:**
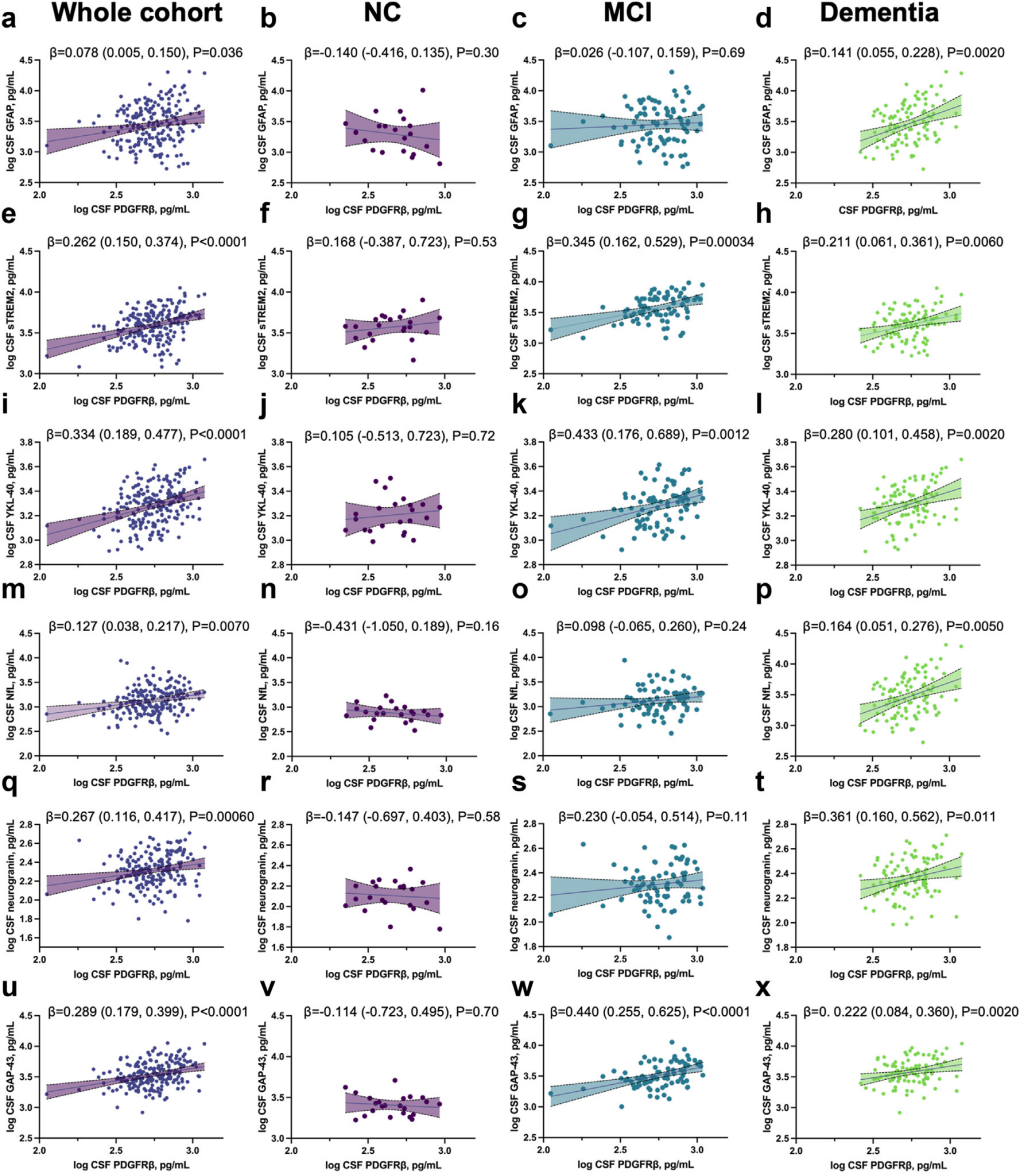
**Association with axonal, synaptic, and neuroinflammation CSF markers**. **a-d** Association of CSF PDGFRβ with CSF GFAP: **a**, in the whole cohort; **b**, in the NC group; **c**, in the MCI group; **d**, in the dementia group. **e-h** Association of CSF PDGFRβ with CSF sTREM 2: **e**, in the whole cohort; **f**, in the NC group; **g**, in the MCI group; **h**, in the dementia group. **i-l** Association of CSF PDGFRβ with CSF YKL-40: **i**, in the whole cohort; **j**, in the NC group; **k**, in the MCI group; **l**, in the dementia group. **m-p** Association of CSF PDGFRβ with CSF NfL: **m**, in the whole cohort; **n**, in the NC group; **o**, in the MCI group; **p**, in the dementia group. **q-t** Association of CSF PDGFRβ with CSF neurogranin: **q**, in the whole cohort; **r**, in the NC group; **s**, in the MCI group; **t**, in the dementia group. **u-x** Association of CSF PDGFRβ with CSF GAP-43: **u**, in the whole cohort; **v**, in the NC group; **w**, in the MCI group; **x**, in the dementia group. Linear regressions adjusting on age, sex, and CSF Aβ42/40 ratio (n = 210). Individual points and the regression line are displayed with a shaded area above and below the line, representing the upper and lower bounds of the 95% confidence interval. Results are shown as unstandardised β estimate (95% confidence interval), P-value.

We tested the correlation between CSF PDGFRβ levels and CSF neuroinflammation, axonal and synaptic markers (Fig. 3). All the CSF neuroinflammation biomarkers were significantly associated with CSF PDGFRβ levels in the whole cohort after adjusting for age, sex, and Aβ ratio (Fig. 3a, e, i). The association was higher for sTREM 2 (β = 0.262 [CI_95%_ 0.150–0.374], P < 0.0001) and YKL-40 (β = 0.334 [CI_95%_ 0.189–0.477], P < 0.0001) than for GFAP (β = 0.078 [CI_95%_ 0.005–0.150]_,_ P = 0.036). Those associations were sustained, focusing on the MCI and dementia groups, but there was no evidence of association in the NC group. Notably, the association with PDGFRβ was significantly higher for CSF GFAP levels in the dementia group (β = 0.141 [CI_95%_ 0.055–0.228], P = 0.0020).

CSF NfL levels were significantly associated with CSF PDGFRβ levels in the whole cohort (β = 0.127 [CI_95%_ 0.038–0.217], P = 0.0060, Fig. 3m) and in the dementia group (β = 0.164 [CI_95%_ 0.051–0.276], P = 0.0050, Fig. 3p).

Regarding CSF synaptic markers (Fig. 3q–x), we observed a significant association of CSF PDGFRβ levels with both GAP-43 levels (β = 0.289 [CI_95%_ 0.179–0.399], P < 0.0001) and neurogranin levels (β = 0.267 [CI_95%_ 0.116–0.417], P = 0.00060). Both significant associations were sustained in the dementia group. At the MCI stage, there was a significant association between PDGFRβ and GAP-43 levels (β = 0.440 [CI_95%_ 0.255–0.625], P < 0.0001), but no evidence of association was found with neurogranin. No evidence of correlation was observed between CSF PDGFRβ and any synaptic marker in the control group.

We performed a principal component analysis to explore the association of CSF PDGFRβ with amyloid, tau, neuroinflammation, axonal, and synaptic CSF markers (Supp. Fig. S4, scree plots Supp. Fig. S5). In the whole cohort, the PCA analysis yielded a ‘core AD’ component (CSF AD biomarkers and synaptic markers, 48% of the variance) while CSF PDGFRβ segregated with neuroinflammation, axonal, and albumin markers in Component 2 (16% of the variance). The analysis in the dementia group yielded similar components. Interestingly, in the MCI group, the PCA analysis yielded 3 components, with a similar ‘core AD’ component 1 (variance = 46%) and a ‘neuroinflammation/axonal’ component 2 (variance = 18%). Notably, CSF PDGFRβ was the only marker in a 3rd component, accounting for 10% of the variance of the biomarkers data.

### Mediation analysis of the effect of PDGFRβ on neurodegeneration and synaptic integrity

**Fig. 4:**
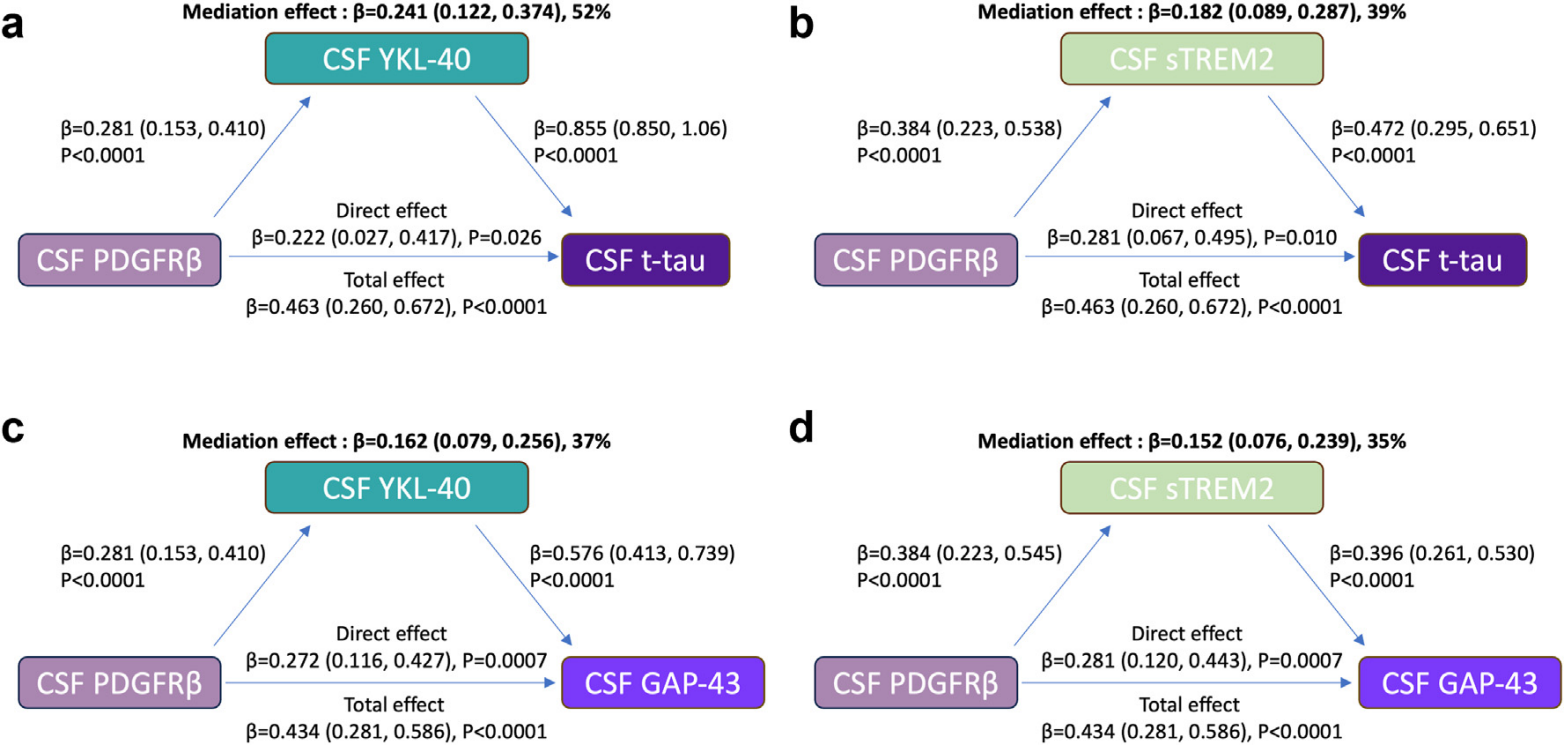
**Mediation analysis of the effect of PDGFRβ on neurodegeneration and synaptic integrity**. Mediation analysis exploring: **a**, CSF YKL-40 as a mediator of the relationship between CSF PDGFRβ and CSF t-tau; **b**, CSF sTREM 2 as a mediator of the relationship between CSF PDGFRβ and CSF t-tau; **c**, CSF YKL-40 as a mediator of the relationship between CSF PDGFRβ and CSF GAP-43; **d**, CSF sTREM2 as a mediator of the relationship between CSF PDGFRβ and CSF GAP-43. Mediation effects are reported as the unstandardised estimate β (95% CI), percentage of the total effect for n = 210. Age, sex and APOE4 status were added as covariates.

To explore the hypothesis that pericyte damage (captured through CSF PDGFRβ) effect on neurodegeneration (reflected by CSF t-tau) and synaptic integrity (reflected by CSF GAP-43 and neurogranin) could be exerted through neuroinflammation (CSF YKL-40, CSF sTREM 2), we performed simple mediation analyses (Fig. 4; model Supp. Fig. S6). In the total population, CSF YKL-40 mediated CSF PDGFRβ effect on CSF t-tau levels (mediation effect, β = 0.241 [CI_95%_ 0.122–0.374], 52% of the total effect; direct effect: β = 0.222 [CI_95%_ 0.027–0.417], P < 0.0001, Fig. 4a). Similarly, CSF sTREM2 mediated CSF PDGFRβ effect on CSF t-tau levels (mediation effect: β = 0.182 [CI_95%_ 0.089–0.287], 39% of total effect; direct effect: β = 0.281 [CI_95%_ 0.067–0.495], P = 0.010, Fig. 4b). CSF YKL-40 mediated the relationship between CSF PDGFRβ and CSF GAP-43 levels (mediation effect: β = 0.162 [CI_95%_ 0.079–0.256], 37% of total effect; direct effect: β = 0.272 [CI_95%_ 0.116–0.427], P = 0.0007, Fig. 4c). CSF sTREM2 mediated in similar proportion the relationship between CSF PDGFRβ and CSF GAP-43 levels (β = 0.152 [CI_95%_ 0.076–0.239], 35% of total effect; direct effect: β = 0.281 [CI_95%_ 0.120–0.443], P = 0.0007, Fig. 4d). Comparable mediation effects were found using CSF neurogranin as a marker of synaptic integrity (Supp. Fig. S7). Mediation analysis was not conducted with CSF GFAP levels as only weak associations were observed with CSF PDGFRβ levels.

## Discussion

There is large evidence of BBB damage in neurodegenerative disorders, with studies highlighting the deleterious effect of proteinopathies, including amyloid, tau, and a-synuclein, on the BBB.^18^^,^^37^ Functional and pathological studies have linked those pathologies to detrimental neuroinflammation at the BBB but also to specific injury to endothelial cells and pericytes. In our work, we found that CSF levels of PDGFRβ were significantly higher at the MCI stage independently of the aetiology (AD or non-AD). Higher PDGFRβ levels were associated with lower cognitive performance, both in MCI and dementia groups. CSF PDGFRβ showed a significant association with CSF p-tau and tau levels but not with the Aβ ratio. CSF PDGFRβ was associated positively with markers of neuroinflammation and synaptic damage. Notably, neuroinflammation markers mediated the relationship between PDGFRβ and markers of neurodegeneration and synaptic integrity.

Accumulating evidence supports the role of BBB disruption in cognitive decline. Prior studies have reported an association of CSF PDGFRβ with cognitive impairment in neurodegenerative disorders, both in cross-sectional and longitudinal studies.^21^^,^^38^ Other fluid markers of the BBB, including albumin quotient and imaging markers, such as enhanced MRI, have also been associated with cognitive decline.^38^^,^^39^ CSF sPDGFRβ was reported to be correlated with dynamic contrast-enhanced MRI markers of BBB dysfunction in the early stage of cognitive dysfunction, with or without Aβ and/or tau changes.^38^ In our PCA analysis, at the MCI stage, PDGFRβ did not segregate with an AD component or a neuroinflammation component but accounted for 10% of the variance as a standalone biomarker. The overall body of evidence suggests that BBB dysfunction starts at an early stage, is associated with cognitive decline, and then plateaus at the mild dementia stage. CSF PDGFRβ levels at the MCI stage could be capturing the specific contribution of pericyte damage to cognitive decline progression.

We observed a weak correlation of CSF PDGFRβ with the CSF/plasma albumin quotient, considered a biomarker of blood-CSF barrier integrity, driven by the dementia group, as it has already been reported.^5^^,^^20^^,^^40^ This could suggest that at dementia stages, pericyte metabolism correlates with increased BBB permeability. However, at earlier stages, pericyte dysfunction might not be directly related to BBB leakiness.

There was no evidence of an association with the CSFAβ42/β40 ratio, considered to be the best CSF correlate of amyloid pathology.^41^ This is in line with the large majority of previous studies, which did not observe an association between CSF PDGFRβ and amyloid pathology measured with PET or in CSF, neither in cognitively unimpaired or impaired subjects.^20^^,^^23^^,^^42^, ^43^, ^44^, ^45^ Notably, no association was found in a cohort of patients with CAA.^43^ We did not observe an association with *APOE* ε4 carriership, a major risk factor for AD, in the total sample, as in previous studies.^20^

Interestingly, we observed a strong association with Aβ40, driven by amyloid-positive subjects, in line with previous studies.^21^^,^^22^^,^^42^^,^^44^ Amyloid β species have been linked to neurovascular dysfunction in ageing and disease.^46^ In cerebral amyloid angiopathy, Aβ40 is the major amyloid species depositing within the walls of cortical and leptomeningeal small vessels.^47^ Some authors have reported an association between Aβ40 immunoreactivity and a reduced number of pericytes in the hippocampus.^48^ Additionally, they observed an aggregation-dependent impact of Aβ40 on pericyte viability and proliferation in vitro. In an electrophysiological study, the contractile response of pericytes was significantly impaired upon exposure to Aβ40 but not to Aβ42.^49^ Thus, this suggests that Aβ40 is a potential regulator of pericyte damage.

Previously published literature consistently supports the association between CSF PDGFR levels and CSF p-tau and t-tau levels.^21^^,^^40^^,^^42^, ^43^, ^44^ In our cohort, the association between CSF PDGFRβ and CSF p-tau and t-tau levels was more marked in amyloid-positive individuals. This association was also reported using tau-PET.^45^ Neuropathological studies have demonstrated the accumulation of oligomeric tau in microvessels in AD and LBD.^50^^,^^51^ Early alterations of the neurovascular unit are observed in mouse and rat models of tauopathy, with altered migration of immune cells.^37^^,^^52^ The association of tau pathology with pericyte alteration could reflect a local clearance failure and/or have a causative effect exerting a detrimental influence on BBB vasculature.

We could not measure markers of α-synuclein pathology, but recent studies have shed light on its relation with pericyte dysfunction in pathological conditions. In cell culture, α-synuclein could induce BBB dysfunction through activation of pericytes releasing inflammatory mediators.^18^ In primary human pericyte cultures, pericytes could degrade *α*-synuclein but succumbed to apoptosis under cellular stress.^53^ Future studies, including patients with Lewy body disease and Parkinson's disease with biomarker evidence of pathology, are needed to characterise the pericyte dysfunction and CSF PDGFRβ metabolism in synucleinopathies.

In our study, CSF PDGFRβ levels were associated with markers of microglial activation YKL-40 and sTREM 2. Microglial cells are a core component of the neurovascular unit, and the interplay between those cells and pericytes is required for a functional BBB.^17^^,^^54^ BBB dysfunction has been associated with neuroinflammation both as a cause and a result.^55^^,^^56^ In our study, YKL-40 and sTREM2 mediated the association of PDGFRβ with markers of synaptic damage (GAP-43 and neurogranin) and neurodegeneration (t-tau). Microglial activation and associated chemokines and oxygen species cascade are known to be detrimental to synapses and neurons.^57^ *In vitro* neuronal cultures and *in vivo* animal models have demonstrated axonal damage and neurodegeneration induced upon inflammatory insults. This supports BBB dysfunction as amplifying neuroinflammation and acting as a key process in the development of synaptic and neuronal loss. In return, the pro-inflammatory role of sTREM2 and YKL-40 in activating microglia may also promote BBB dysfunction as the microglia is implicated in regulating core mechanisms for BBB, such as recruiting peripheral immune cells and safeguarding the integrity of tight junction proteins.^58^ Interestingly, the association observed between CSF PDGFRβ levels and CSF YKL-40 and sTREM2 was independent of markers of amyloid pathology, which has already been described.^59^ It has been hypothesised that, after a very early amyloid-driven microglial association, the microglial activation independently continues along disease progression. Overall, the microglial activation at the BBB most likely exerts a dual effect, with a maintenance role, but also impairing BBB function during sustained neuroinflammation and thus promoting neurodegeneration.^60^

We found a weaker association of CSF PDGFRβ levels with CSF GFAP levels, sometimes confounded by CSF amyloid levels, although there is robust evidence for the interaction of pericytes and astrocytes at the BBB, both in physiological and pathological functions.^61^ Reactive gliosis is a common feature of astrocytes during BBB disruption, with a detrimental effect on the BBB function and subsequent damage in neuronal survival. In a transcriptomic brain tissue study, significant transcriptome changes were detected in the AD brain at the gliovascular unit, associated primarily with impaired pericyte-to-astrocyte interactions.^62^ However, an important element to consider is that CSF and plasma GFAP have been reported to be very strongly associated with markers of amyloid pathology.^31^ Thus, by measuring CSF GFAP levels, we might be capturing an amyloid-driven astrocytic activation more than the astrocytic processes taking place at the BBB.

Besides the detrimental effect of the BBB disruption accompanying pericyte dysfunction and neuroinflammation, modification in PDGFRβ signalling could directly affect neurons and synapses. PDGF signalling, including PDGFRβ, has been implicated in adult neuronal maintenance in vitro and mouse models.^63^ Notably, PDGFRβ was found to exert neuroprotective effects upon injury, limiting excitotoxicity and apoptosis. Reduced expression of post-synaptic marker expression and impairment in hippocampal long-term potentiation were observed in PDGFRβ knockout mice.^64^ In hippocampal neuron cultures, amyloid β inhibited PDGFβ receptor phosphorylation and attenuated the ability of PDGF to protect neurons against excitotoxicity.^65^ Thus, alterations of pericyte signalling likely exert detrimental effects on neuronal and synaptic functioning through both direct and indirect mechanisms.

### Caveats and limitations

Our study is not without limitations. We did not measure any markers of synucleinopathy, which are now available in the form of alpha-synuclein seed amplification assays. However, as validated biomarkers for non-amyloid and non-tau pathologies are still lacking, we included well-phenotyped individuals meeting the most recent clinical diagnosis for non-AD dementia. Our sample was of moderate size and included mostly patients on the AD continuum, with a limited number of non-AD patients and controls. Thus, it should indicate some caution in our interpretation of our results for specific diagnosis groups, such as DLB or FTD. Future directions include replication of our findings in larger multicentre cohorts, including non-AD dementia. Although we have adjusted all analyses for age and sex, residual confounding could also have affected our overall results, such as small vessel disease or anti-inflammatory medication. Similarly, we did not stratify our mediation analysis by APOE status or diagnosis nor included other potential confounders other than age and sex. Future studies with larger sample sizes are needed to make accurate conclusions regarding APOE4 role or other potential confounder to further characterise the relation between BBB impairment and neuroinflammation.

We performed a cross-sectional study; longitudinal studies will be needed to inform the temporality of pericyte dysfunction along disease progression and its association with cognitive decline.

## Conclusion

We found that CSF PDGFR β levels were associated with alterations in CSF biomarkers related to synaptic function, axonal integrity, and neuroinflammation, alongside markers of neurodegeneration in degenerative conditions. Pericyte dysfunction appeared to be a significant factor in BBB dysfunction and cognitive impairment at the MCI stage, independently of amyloid or tau changes. Mediation analysis indicated that neuroinflammation might mediate the adverse impact of pericyte dysfunction on synaptic integrity and contribute to neurodegeneration. Further studies will be needed to elucidate the interplay between pericyte dysfunction and neuroinflammation. Better characterisation of the processes occurring at the BBB could contribute to the understanding of the variability in disease progression and cognitive decline and, potentially, explain differences in therapeutic response in neurodegenerative disorders.

## Contributors

Study concept or design: AV, NJA, HZ, KB, CP; acquisition of data: AV, NJA, TKK, JD, EC, EBA, CP; analysis or interpretation of data: AV, NJA, FML, VP, HZ, KB, CP; drafting/revision of the manuscript for content: all authors. All authors read and approved the final version of the manuscript. AV and CP have accessed and verified the data.

## Data sharing statement

The anonymised dataset used in the current study is available from the corresponding author upon reasonable request (agathe.vrillon@aphp.fr), after approval of a proposal.

## Declaration of interests

ML has participated in an educational program for Eisai, unrelated to this work. HZ has served on scientific advisory boards for Alector, Eisai, Denali, Roche Diagnostics, Wave, Samumed, Siemens Healthineers, Pinteon Therapeutics, Nervgen, AZTherapies, and CogRx, has given lectures in symposia sponsored by Cellectricon, Fujirebio, Alzecure and Biogen, and is a co-founder of Brain Biomarker Solutions in Gothenburg AB (BBS), which is a part of the GU Ventures Incubator Program (outside submitted work), all unrelated to this work. TKK was supported by the NIH (R01 AG083874, U24 AG082930) and the Alzheimer's Association (#AARF-21-850325) and has received honoraria from the University of Wisconsin Madison and the University of Pennsylvania and has an awarded patent (#WO2020193500A1), all unrelated to this work. KB has served as a consultant and on advisory boards for Acumen, ALZPath, BioArctic, Biogen, Eisai, Lilly, and Moleac Pte. Ltd, Novartis, Ono Pharma, Prothena, Roche Diagnostics, and Siemens Healthineers; has served on data monitoring committees for Julius Clinical and Novartis; has given lectures, produced educational materials, and participated in educational programs for AC Immune, Biogen, Celdara Medical, Eisai and Roche Diagnostics; and is a co-founder of Brain Biomarker Solutions in Gothenburg AB (BBS), which is a part of the GU Ventures Incubator Program (outside submitted work), all unrelated to this work. CP is a member of the International Advisory Boards of Lilly; is a consultant for Fujirebio, Alzhois, Neuroimmune, Ads Neuroscience, Roche, AgenT, and Gilead; and is involved as an investigator in several clinical trials for Roche, Esai, Lilly, Biogen, Astra-Zeneca, Lundbeck, and Neuroimmune, all unrelated to this work.

## Appendix A Supplementary data

Supplementary Figures and Tables

CPP 2023
