# Supplementary material for: Dissection of blood–brain barrier dysfunction through CSF PDGFRβ and amyloid, tau, neuroinflammation, and synaptic CSF biomarkers in neurodegenerative disorders
Source: eBioMedicine. 2025 Apr 15;115:105694. doi: 10.1016/j.ebiom.2025.105694 (PMC12020895; doi:10.1016/j.ebiom.2025.105694)
Supplement: Supplementary Figures and Tables [file mmc1.docx]

**SUPPLEMENTARY MATERIALS**

**Supplementary Table 1, Cohort characteristics and biomarker levels by diagnosis**

**Supplementary Table 2, Cohort characteristics and biomarker levels by sex**

**Supplementary Table 3, Association with age, sex, and *APOE4* carriership**

**Supplementary Figure 1, Directed acyclic graph to identify the minimally sufficient set of measured confounders to be adjusted for the exploration of the association of CSF PDGFRβ with cognitive status**

**Supplementary Figure 2, Plasma PDGFRβ and PDGFRβ ratio levels and association with CSF PDGFRβ**

**Supplementary Figure 3, CSF PDGFRβ levels across AT(N) status at MCI and dementia stages**

**Supplementary Figure 4, PCA analysis of CSF biomarkers in the whole cohort, MCI and dementia groups**

**Supplementary Figure 5, Principal component analysis scree plots**

**Supplementary Figure 6, Mediation model**

**Supplementary Figure 7, Mediation analysis of the effect of CSF neuroinflammation markers on CSF PDGFRβ association with CSF neurogranin**

|  | **NC** | **AD-MCI** | **AD dementia** | **Non-AD MCI** | **Non-AD dementia** | **P-value*** |
| --- | --- | --- | --- | --- | --- | --- |
|  | **n=23** | **n=41** | **n=73** | **n=43** | **N=30** |  |
| Age, years | 61.0 [57.0-69.0] | 74.0 [67.5-79.0] | 72.0 [66.5-79.5] | 68.0 [62.0-74.0] | 68.0 [58.7-71.0] | 0.00012 |
| Sex, male | 39% (9) | 37% (15) | 37% (27) | 37% (16) | 50% (15) | 0.50 |
| *APOE* ε4 carriership | 39% (9) | 59% (24) | 68% (50) | 12% (5) | 27% (8) | <0.0001 |
| MMSE | 28 [26-30] | 25 [22-27] | 19 [16-24] | 25 [22-27] | 25 [19-27] | <0.0001 |
| LoE, years | 15 [12-15] | 15 [11-15] | 11 [7-15] | 11 [11-15] | 15 [11-15] | 0.00034 |
| **AD CSF biomarkers** |  |  |  |  |  |  |
| CSF Aβ40, pg/mL | 11706 [10514-13548] | 12794 [9013-15163] | 11597 [9021-14978] | 11775 [8520-14134] | 10354 [8963-12884] | 0.50 |
| CSF Aβ42, pg/mL | 1036 [925-1253] | 518 [379-629] | 480 [384-606] | 1101 [673-1327] | 887 [677-1206] | <0.0001 |
| CSFAβ42/40 ratio | 0.094 [0.087-0.099] | 0.042 [0.036-0.052] | 0.041 [0.035-0.047] | 0.092 [0.081-0.097] | 0.091 [0.081-0.099] | <0.0001 |
| CSF p-tau181, pg/mL | 33.4 [26.5-39.1] | 80.1 [62.7-107.9] | 99.8 [70.4-144.2] | 35.3 [26.2-49.4] | 33.05 [28.4-45.1] | <0.0001 |
| CSF t-tau, pg/mL | 226 [193-291] | 526 [417-664] | 623 [447-940] | 302 [199-384] | 285 [191-374] | <0.0001 |
| **ATN status** |  |  |  |  |  | <0.0001 |
| A-T- | 100% (23) | - | - | 93% (40) | 80% (24) |  |
| A+T- | - | 22% (9) | 11% (8) | - | 10% (3) |  |
| A+T+ | - | 78% (32) | 89% (65) | - | - |  |
| A-T+ | - | - | - | 7% (3) | 10% (3) |  |
| **BBB biomarkers** |  |  |  |  |  |  |
| CSF albumin, pg/mL | 271 [195-317] | 296 [216-328] | 222 [184-322] | 245.5 [211-305] | 272.5 [179-326] | 0.21 |
| Plasma albumin, pg/mL | 44.2 [39.9-46.8] | 41.7 [40.1-44.0] | 41.7 [39.1-43.7] | 41.5 [38.4-43.1] | 41.9 [38.9-45.0] | 0.27 |
| Albumin quotient | 0.171 [0.127-0.238] | 0.139 [0.113-0.188] | 0.185 [0.135-0.223] | 0.158 [0.127-0.197] | 0.147 [0.121-0.222] | 0.10 |
| Plasma PDGFRβ, pg/mL | 7.50 [5.64-9.42] | 8.82 [7.43-10.6] | 7.47 [6.41-11.1] | 9.23 [8.05-10.9] | 8.27 [7.13-16.5] | 0.12 |
| CSF PDGFRβ, pg/mL | 474 [340-596] | 566 [426-724] | 549 [434-698] | 596 [460-823] | 515 [438-616] | 0.096 |
| Plasma/CSF PDGFRβ quotient | 17.8 [12.0-26.0] | 15.1 [11.4-19.4] | 15.2 [10.6-20.0] | 17.4 [11.8-23.4] | 16.37 [11.2-32.7] | 0.64 |

**Supplementary Table 1, Cohort characteristics and biomarker levels by diagnosis**

Continuous variables are presented as median [IQR] and categorical data as number (%). *Age, MMSE scores, and levels of education were compared between groups using the Kruskal-Wallis test. *APOE* ɛ4 carriership, sex and AT(N) group frequencies were compared between groups using Fisher's test. Biomarkers levels were compared across groups using one-way ANCOVA adjusting for age and sex.

Abbreviations: Aβ, amyloid beta; *APOE*, apolipoprotein E; BBB, brain blood barrier; CSF, cerebrospinal fluid; LoE, levels of education; MCI, mild cognitive impairment; MMSE, mini-mental state examination; NC, neurological controls; PDGFRβ, Platelet derived growth factor receptor beta ; p-tau181, tau phosphorylated at serine 181

|  | NC | | MCI | | Dementia | |
| --- | --- | --- | --- | --- | --- | --- |
|  | **Female** | **Male** | **Female** | **Male** | **Female** | **Male** |
|  | **n=14** | **n=9** | **n=53** | **n=31** | **n=61** | **n=42** |
| Age, years | 62.0 [52.0, 81.0] | 61.0 [47.0, 78.0] | 68.0 [43.0, 84.0] | 74.0 [45.0, 85.0] | 70.0 [52.0, 84.0] | 72.0 [51.0, 87.0] |
| APOE ε4 carriership | 5 (35.7%) | 4 (44.4%) | 20 (37.7%) | 9 (29.0%) | 36 (59.0%) | 22 (52.4%) |
| MMSE | 28 [21, 30] | 28 [26, 30] | 25 [9, 29] | 26 [11, 30] | 20.0 [5, 29] | 20 [11, 29] |
| Level of education | |  |  |  |  |  |
| Below high school | 1 (7.1%) | 1 (11.1%) | 6 (11.3%) | 3 (9.7%) | 18 (29.5%) | 8 (19.0%) |
| Above high school | 10 (71.4%) | 8 (88.9%) | 43 (81.1%) | 26 (83.9%) | 37 (60.7%) | 29 (69.0%) |
| Non available | 3 (21.4%) | 0 (0%) | 4 (7.5%) | 2 (6.5%) | 6 (9.8%) | 5 (11.9%) |
| AD CSF biomarkers | |  |  |  |  |  |
| CSF Aβ40, pg/mL | 11800 [7640, 17800] | 10600 [7950, 14700] | 11700 [3270, 29100] | 12900 [6220, 25500] | 11100 [6520, 21600] | 11800 [4780, 19200] |
| CSF Aβ42, pg/mL | 1100 [759, 1830] | 1010 [660, 1600] | 606 [235, 2340] | 775 [370, 2340] | 518 [257, 1570] | 587 [215, 1590] |
| CSFAβ42/40 ratio | 0.0930 [0.0870, 0.104] | 0.0950 [0.0620, 0.109] | 0.0620 [0.0290, 0.103] | 0.0650 [0.0320, 0.106] | 0.0440 [0.0260, 0.106] | 0.0495 [0.0230, 0.110] |
| CSF p-tau181, pg/mL | 34.3 [18.6, 45.7] | 31.0 [23.2, 44.3] | 54.0 [10.9, 173] | 60.0 [21.4, 272] | 89.3 [21.4, 285] | 66.0 [17.0, 228] |
| CSF t-tau, pg/mL | 238 [158, 379] | 214 [146, 326] | 392 [105, 1050] | 413 [125, 1630] | 580 [154, 2000] | 414 [124, 1320] |
| ATN status |  |  |  |  |  |  |
| A-T- | 14 (100%) | 9 (100%) | 25 (47.2%) | 15 (48.4%) | 11 (18.0%) | 13 (31.0%) |
| A-T+ | - | - | 2 (3.8%) | 1 (3.2%) | 3 (4.9%) | - |
| A+T- | - | - | 7 (13.2%) | 2 (6.5%) | 6 (9.8%) | 5 (11.9%) |
| A+T+ | - | - | 19 (35.8%) | 13 (41.9%) | 41 (67.2%) | 24 (57.1%) |
| Blood brain barrier biomarkers | | |  |  |  |  |
| CSF PDGFRβ | 417 [226, 761] | 539 [263, 925] | 552 [111, 1090] | 631 [243, 1060] | 549 [263, 1190] | 531 [284, 1100] |
| Plasma PDGFRβ | 7480 [4460, 19900] | 9110 [3600, 49700] | 9740 [3410, 21600] | 8790 [4860, 16400] | 7760 [3010, 47700] | 8050 [2720, 24600] |
| Ratio plasma/CSF PDGFRβ | 17.8 [8.33, 45.1] | 16.0 [10.5, 83.5] | 16.1 [7.46, 79.2] | 13.7 [6.50, 111] | 15.7 [5.42, 98.2] | 15.9 [3.93, 45.4] |
| Q-Alb | 5.53 [2.77, 8.85] | 7.33 [2.67, 13.0] | 6.36 [2.48, 22.7] | 7.24 [3.22, 19.9] | 5.01 [2.55, 14.6] | 6.89 [2.42, 15.0] |

**Supplementary Table 2, Cohort characteristics and biomarker levels by sex**

Continuous variables are presented as median [IQR] and categorical data as number (%).

Abbreviations: Aβ, amyloid beta; *APOE*, apolipoprotein E; BBB, brain blood barrier; CSF, cerebrospinal fluid; LoE, levels of education; MCI, mild cognitive impairment; MMSE, mini-mental state examination; NC, neurological controls; PDGFRβ, Platelet derived growth factor receptor beta; p-tau181, tau phosphorylated at serine 181.

|  |  | **Whole cohort** | **NC** | **MCI** | **Dementia** |
| --- | --- | --- | --- | --- | --- |
| Age | Unadjusted | r= 0.221 (0.0870, 0.350)  P=0.0010 | r=-0.030 (-0.451, 0.389)  P=0.89 | r=0.151 (-0.071, 0.361)  P=0.17 | r=0.272 (0.081, 0.454)  P=0.0050 |
|  | Adjusted on sex and *APOE* | β=0.436 (0.087, 0.784) P=0.015 | β= -0.311 (-1.312, 0.690) P=0.52 | β=0.121 (-0.504, 0.747) P=0.70 | β=0.632 (0.158, 1.105) P=0.0090 |
| Sex | Unadjusted | P=0.098 | P=0.28 | P=0.14 | P=0.62 |
|  | Adjusted on age and *APOE* | β= -0.031 (-0.074, 0.012) P=0.16 | β=-0.096 (-0.224, 0.033) P=0.13 | β=-0.050 (-0.128, 0.028) P=0.20 | β=-0.08 (-0.061, 0.045) P=0.75 |
| *APOE* ε4 carriership | Unadjusted | P=0.17 | P=0.033 | P=0.027 | P=0.33 |
|  | Adjusted on age and sex | β= -0.030 (-0.072, 0.011) P=0.15 | β=-0.164 (-0.295, -0.034) P=0.016 | β=-0.068 (-0.145, 0.010) P=0.087 | β=0.019 (-0.034, 0.071) P=0.48 |

**Supplementary Table 3, Association with age, sex, and *APOE* ε4 carriership**

Association of CSF PDGFRβ with age, sex, and *APOE* ε4 carriership were studied unadjusted using Spearman's correlation for age and Chi-2 test for sex and *APOE* ε4 carriership, and with linear regression for adjusted analysis. Results are presented as unstandardized β estimates (95% confidence interval) and P-value.

**Supplementary Figure 1, Directed acyclic graph to identify the minimally sufficient set of measured confounders to be adjusted for the exploration of the association of CSF PDGFRβ with cognitive status**

**Supplementary Figure 2, Plasma PDGFRβ and PDGFRβ ratio levels and association with CSF PDGFRβ**

**a**, Plasma PDGFRβ levels across diagnosis groups; **b**, CSF/plasma PDGFRβ ratio across diagnosis groups; **c**, Plasma PDGFRβ levels across syndrome groups; **d**, CSF/plasma PDGFRβ ratio across syndrome groups. P-values were obtained through one-way ANCOVA adjusting for age and sex, followed by post hoc Tukey's test, adjusting for multiple comparisons. Box and whiskers plots with the central line denoting median value, and the box containing the 25^th^ to 75^th^ percentile values.

Correlation of CSF PDGFRβ with plasma: **e**, in the whole sample; **f**, neurological controls; **g**, MCI; and **h**, dementia group. The association between biomarkers was studied using Spearman's rank correlation. Individual points and the regression line are displayed with a shaded area above and below the line, representing the upper and lower bounds of the 95% confidence interval. Results are presented as r (95% confidence interval) and P-values.

**Supplementary Figure 3, CSF PDGFRBeta levels across AT(N) status at MCI and dementia stages**

CSF PDGFRβ levels across AT(N) groups in: **a**, in the MCI; **b**, dementia subgroup. CSF PDGFRβ levels were compared using one-way ANCOVA adjusting for age and sex. Effect sizes were estimated with Cohen’s *d*. Box and whiskers plots with the central line denoting median value and the box containing the 25^th^ to 75^th^ percentile values.

**Supplementary Figure 4, PCA analysis of CSF biomarkers in the whole cohort, MCI, and dementia groups**

Principal component analysis of CSF biomarkers in **a,** the whole cohort; **b,** the dementia group; and **c,** the MCI group. CSF biomarker values were log-transformed and z-scored before analysis. The adequacy of the sample was verified using Kaiser-Meyer-Olkin (KMO) measure of sampling adequacy (Whole cohort, KMO=0.841; dementia, KM0=0.805; MCI, KMO=0.827) and Bartlett's Test of Sphericity (all, P<0.001). The number of components was determined by the number of eigenvalues greater than one. The percentage of variance explained by the component is displayed on each graph axis. The sample in the NC group did not meet the sample adequacy criteria to perform the analysis.

**a, Whole cohort**

**b, MCI group**

**c, Dementia group**

**Supplementary Figure 5, Principal component analysis scree plots**

Scree plots of factors extracted in principal component analysis for **a,** the whole cohort; **b,** in the MCI subgroups; and **c,** in the dementia subgroup. We considered components with eigenvalues superior to 1.

**Supplementary Figure 6, Mediation model**

**Supplementary Figure 7, Mediation analysis of the effect of CSF neuroinflammation markers on CSF PDGFRβ association with CSF neurogranin**

Mediation analysis in the whole cohort exploring: **a**, CSF YKL-40 as a mediator of the relationship between CSF PDGFRβ and CSF neurogranin; **b,** CSF sTREM2 as a mediator of the relationship between CSF PDGFRβ and CSF neurogranin.

Indirect effects are reported as the unstandardized estimate β (95% CI), percentage of the total effect.
