# Supplementary material for: Dissection of blood–brain barrier dysfunction through CSF PDGFRβ and amyloid, tau, neuroinflammation, and synaptic CSF biomarkers in neurodegenerative disorders
Source: eBioMedicine. 2025 Apr 15;115:105694. doi: 10.1016/j.ebiom.2025.105694 (PMC12020895; doi:10.1016/j.ebiom.2025.105694)

Comité de Protection des Personnes Est III
Avis sur une demande initiale
Référence CPP 23.10.02

CPP

Nom du CPP : Comité de Protection des Personnes Est III
Adresse : CHRU de Nancy - Bâtiment principal - Rez de chaussée - Rue du Morvan 54511 VANDOEUVRE-LES-NANCY France
Courriel : cppest.3@chru-nancy.fr
Téléphone : 0383154324

Promoteur / Demandeur

Promoteur : APHP-DRCI-URC Lariboisière-Fernand Widal-Saint-Louis (site Saint-Louis)
Représentant légal (UE) : -
Mandataire : -

Dossier

Numéro SI : 23.03012.000249
Numéro national : 2023-A01413-42
Référence interne : APHP230999

Règlementation : Loi Jardé
Qualification : Catégorie 2
Produit ou acte : Hors produits de santé (produits non mentionnés à l'article L.5311-11 du code de la santé publique)

Investigateur : Pr Claire PAQUET

Titre : **« Collection biologique des atteintes neurocognitives » BioCogBank**

Extrait des délibérations du 3 octobre 2023 du Comité de Protection des Personnes EST III, dont la séance non publique s'est tenue à cette date à compter de 16 heures, sur convocation de ses membres par la Présidente.

Aucun conflit d'intérêt direct ou indirect avec le Promoteur ou l'investigateur coordonnateur au sens de l'article L.1123-3 n'a été identifié.

Le quorum a bien été atteint après comptage des membres présents.

Siégeaient ensembles ce 3 octobre 2023, sous la Présidence de Madame le Docteur Elisabeth LUPORSI,

MEMBRES DU COLLEGE 1
Médecins ayant une qualification et une expérience approfondie en matière de RIPH

- Professeur Gérard AUDIBERT
- Professeur Thomas LECOMPTE
- Docteur Elisabeth LUPORSI
- Docteur Nathalie WIRTH

Médecins Généralistes
- Docteur Dominique CHONE
- Docteur Patrick PETON

Pharmaciens Hospitaliers
- Docteur Marie SOCHA

Auxiliaires Médicaux
- Monsieur Guillaume PFEIFFER

MEMBRES DU COLLEGE 2

Compétences à l'égard des questions éthiques
- Madame Huguette MAUSS
- Professeur Yves MARTINET

Compétences en sciences humaines ou sociales
- Madame Hélène HUMBERT

Compétences en matière juridique
- Madame Sophie ZEVACO

Représentants des associations agréées de malades ou d'usagers du système de santé
- Madame Laurence HEBTING-MANACHE
- Madame Séverine JUPPONT

Ce dossier a été étudié en séance le 03/10/2023 et mandat a été donné au président du CPP d'émettre l'avis à réception des réponses du déposant aux dernières demandes. Au vu des réponses obtenues le 23/10/2023, l'avis suivant a donc été émis. Cet avis court à compter du changement de statut sur le SI.

Considérant que les conditions éthiques sont remplies notamment au regard des éléments de l'article L.1123-7 du code de la santé publique, l'examen du comité permet de conclure que la recherche peut être réalisée et de rendre l'avis suivant :

Avis favorable

Cet avis est valable deux ans. Conformément à l'article L.1123-11 du code de la santé publique, le promoteur doit déclarer au CPP le début de la recherche. Cette déclaration se fait directement sur le SIRIPH2G (bouton "démarrer l'étude").

Si vous n'avez pas été en mesure d'inclure un premier participant à la recherche dans ce délai, vous pouvez demander au CPP une prorogation de cet avis avant la fin de validité de ce dernier (article R.1123-26 du code de la santé publique).

Documents analysés par le CPP

Catégorie	Intitulé	Date de dépôt
ADD - Doc additionnel	*2023-A01413-42_document-additionnel-_V1.0_20230822_BioCogBank.pdf*	28/08/2023
ASS - Assurance	*2023-A01413-42_Attestation d'assurance_APHP230999_BioCogBank.pdf*	28/08/2023
COU - Courrier	*2023-A01413-42_Courrier à l'attention du CPP_V1.0_20230822_BioCogBank.pdf*	28/08/2023
CVI - CV investigateurs	*2023-A01413-42 _CV INVESTIGATEURS_v1.0_20230524_BiocogBank.pdf*	28/08/2023
DEM - Demande autorisation	*2023-A01413-42_Formulaire demande initiale_HPS_RIPH2_20230822_BioCogBank.pdf*	28/08/2023
DOC - Autres documents	*2023-A01413-42_MR-001_declaration-conformite_20230120_BioCogBank.pdf*	28/08/2023
DOC - Autres documents	*2023-A01413-42- ATTESTATIONS BPC_ v1_20230524_BioCogBank.pdf*	05/09/2023
INF - Doc Information	*2023-A01413-42_NIFC_V1.0_20230524_BioCogBank.docx*	12/09/2023
JUS - Justification lieux de recherche	*2023-A01413-42_Justif-adequation-moyens-HPS_20230822_BioCogBank.pdf*	28/08/2023
PRO - Protocole	*2023-A01413-42_protocole_V1.1_20231012_BioCogBank_version suivi de modification.docx*	23/10/2023
PRO - Protocole	*2023-A01413-42_protocole_V1.1_20231012_BioCogBank_version finale.pdf*	23/10/2023
REP - Courrier de réponse	*2023-A01413-42_COURRIER-reponse CPP_v1.0_20231012_BioCogBank.pdf*	23/10/2023
RES - Résumé	*2023-A01413-42_RESUME SYNOPTIQUE_V1.0_20230524_BioCogBank.docx*	12/09/2023

**Les documents étiquetés non-conformes sur le SI RIPH2G ou transmis pour information/notification dans le cadre de cette demande d'avis n'ont pas été évalués par le CPP.*
**L'intitulé des documents examinés par le comité, listés sur le présent avis, reprend la nomenclature des fichiers utilisée par le déposant sur le SI RIPH2G.*

Le mercredi 25 Octobre 2023

La Présidente du CPP EST III
Madame le Docteur Elisabeth LUPORSI

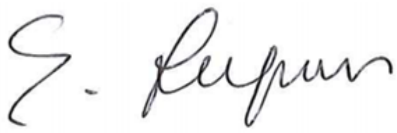

Supplement: CPP 2023 [file mmc2.pdf]
